# Rivaroxaban alleviates hepatic sinusoidal obstruction syndrome in mice by modulating the gut microbiota and inhibiting the PI3K/Akt signaling pathway

**DOI:** 10.3389/fmicb.2025.1607131

**Published:** 2025-08-21

**Authors:** Wencheng Liu, Yanbin Cheng, Xue Han, Junlin Xia, Qingyu Wei, Bing Chang, Quansheng Li

**Affiliations:** ^1^Department of Gastroenterology, The First Affiliated Hospital of China Medical University, Shenyang, Liaoning, China; ^2^Department of Cardiovascular Ultrasound, The First Hospital of China Medical University, Shenyang, Liaoning, China; ^3^Department of Medical Basic Experimental Teaching Center, China Medical University, Shenyang, Liaoning, China; ^4^Department of Allergy, Shengjing Hospital of China Medical University, Shenyang, Liaoning, China

**Keywords:** rivaroxaban, hepatic sinusoidal obstruction syndrome, gut microbiota, transcriptomic analysis, liver injury

## Abstract

**Introduction:**

Hepatic sinusoidal obstruction syndrome (HSOS) is a vascular liver disease with a high mortality rate, and treatment methods are limited. Rivaroxaban is an oral anticoagulant. This study aimed to investigate the pharmacological effect and potential mechanism of rivaroxaban on HSOS.

**Methods:**

In this study, we induced an HSOS mouse model in male C57BL/6J mice by administering monocrotaline orally. The mice were randomly divided into four groups: the control group, the rivaroxaban (RIV) group, the monocrotaline (MCT) group, and the monocrotaline + rivaroxaban (MCT + RIV) group. Liver function and histopathology were evaluated. 16S rDNA sequencing of the small intestinal contents, transcriptomic sequencing of small intestine tissues, real-time qPCR, Western blot analysis of liver tissues, and correlation analysis were conducted. Antibiotic (ABX) treatment and fecal microbiota transplantation (FMT) experiments were also performed to explore the role of the gut microbiota.

**Results:**

Compared with the MCT group, rivaroxaban alleviated serum biochemical liver function analysis and liver histopathology in the MCT + RIV group. Additionally, 16S rDNA sequencing of the small intestinal contents revealed that, compared with the MCT group, the MCT + RIV group presented increased relative abundances of Allobaculum and Pediococcus but decreased relative abundances of Streptococcus, Staphylococcus, and Candidatus Arthromitus. Mechanistically, integrated analyses, including transcriptomic sequencing of small intestin e tissues, real-time qPCR, Western blot analysis of liver tissues, and correlation analysis, demonstrated that rivaroxaban protected against MCT-HSOS by inhibiting the PI3K/Akt signaling pathway. In addition, antimicrobial cocktail (ABX) treatment eliminated the beneficial effects of rivaroxaban on liver function and histopathological injury, whereas fecal microbiota transplantation (FMT) from rivaroxaban-treated donors significantly ameliorated liver dysfunction and histological damage in MCT-HSOS mice.

**Discussion:**

These findings suggest that rivaroxaban alleviates hepatic sinusoidal obstruction syndrome in mice by modulating the gut microbiota and inhibiting the PI3K/Akt signaling pathway. Rivaroxaban may be a promising therapeutic option for treating HSOS.

## 1 Introduction

Hepatic sinusoidal obstruction syndrome (HSOS) is a hepatic vascular condition resulting from endothelial cell damage and subsequent obstruction of the hepatic sinusoids. Ascites, abdominal distention, hepatomegaly, and jaundice are the primary clinical symptoms associated with HSOS (Zhuge et al., 2019). The liver histological abnormalities observed in HSOS under the microscope include several alterations, such as damage to and dissection of hepatic sinusoidal endothelial cells, coagulative necrosis and hemorrhage in the centrilobular region, and aggregation of monocytes (DeLeve et al., 2003). In developed countries, cytoreductive therapy prior to hematopoietic stem cell transplantation (HSCT) and oxaliplatin-containing chemotherapy are the primary causes of HSOS (Yang et al., 2019). In China, the primary factor contributing to HSOS is the consumption of Tu-San-Qi (*Gynura segetum* or *Gynura japonica*), which contains pyrrolizidine alkaloids (PAs) (Zhu et al., 2021). Depending upon the risk factors present, the mean prevalence of HSCT-HSOS is 14% (range 0–60%) (Dignan et al., 2013). However, the average prevalence of PA-HSOS is still uncertain.

Individuals suffering from severe HSOS frequently experience multiorgan failure and have a mortality rate above 80% (Coppell et al., 2010; Zhang et al., 2019). As a result, this leads to increased utilization of medical resources, which imposes a significant medical burden. Moreover, the treatment methods and effects of HSOS are still limited in clinical practice. Research has demonstrated that the administration of anticoagulation agents mitigates clinical symptoms and enhances the survival of individuals diagnosed with mild or moderate HSOS (Peng et al., 2020). The combination of low-molecular-weight heparin and warfarin has an efficacy rate of 60.3% in treating HSOS (Zhuge et al., 2018). Nevertheless, the above anticoagulant drugs have a high risk of bleeding, and their effectiveness needs to be improved. Therefore, further investigations of potential disease mechanisms and the development of novel therapeutic approaches are necessary.

Rivaroxaban functions as an oral anticoagulant by inhibiting factor Xa (Samama, 2011; Beyer-Westendorf et al., 2017). Compared with warfarin treatment, rivaroxaban has been shown to reduce major bleeding in studies evaluating the management of atrial fibrillation in individuals with heart failure (Jackevicius et al., 2021). In research on venous thromboembolism, rivaroxaban has demonstrated comparable efficacy and a lower likelihood of bleeding compared with low-molecular-weight heparin or vitamin K therapy (Chan et al., 2020). Consequently, rivaroxaban has great potential as a specialized treatment for HSOS because of its high level of safety and effectiveness. Nevertheless, the mechanism remains incompletely understood, necessitating additional investigation.

A substantial body of research indicates that dysbiosis of the gut microbiota plays a crucial role in the development of liver disorders. Hepatic carbohydrate and lipid metabolism in individuals with non-alcoholic fatty liver disease (NAFLD) is influenced by the gut microbiota, which can lead to the progression of liver inflammation (Kolodziejczyk et al., 2019). Concurrently, NAFLD is associated with elevated levels of intestinal Gram-negative bacteria, such as *Bacteroidaceae* and *Enterobacteriaceae*, which consequently exacerbate liver fibrosis (De Minicis et al., 2014). Currently, evidence from clinical research suggests that the administration of probiotics, symbiotics, and fecal microbiota transplantation can effectively regulate the gut microbiota composition in individuals, resulting in a reduction in NAFLD (Nobili et al., 2019). Additionally, certain therapeutic interventions have demonstrated efficacy in alleviating liver diseases by modulating the gut microbiota. Berberine has the potential to alleviate non-alcoholic steatohepatitis (NASH) by modulating the population of lipid metabolism-associated bacteria, such as those in the *Bacteroidaceae* family and *Bacteroides* genus (Li et al., 2022). By decreasing the relative abundance of bacteria associated with obesity and metabolic conditions, such as *Alistipes, Anaerotruncus*, and *Desulfovibrio*, and increasing the relative number of bacteria that produce short-chain fatty acids (SCFAs), such as *Lactobacillaceae* and *Ruminococcaceae*, diammonium glycyrrhizinate alleviated NAFLD (Li et al., 2018). Therefore, rivaroxaban has the potential to treat HSOS through its effects on the gut microbiota. To date, reports on the potential associations between the development of HSOS and the gut microbiota, as well as the potential impact of rivaroxaban on HSOS via the gut microbiota, are lacking.

Therefore, our study aimed to investigate the effects of rivaroxaban on MCT-induced HSOS and its underlying mechanism. This study demonstrated that rivaroxaban significantly ameliorates hepatic injury in MCT-induced HSOS (MCT-HSOS) mice through two independent mechanisms: modulating the gut microbiota composition and inhibiting the PI3K/Akt signaling pathway. Furthermore, correlation analysis revealed a potential association between alterations in gut microbiota and suppression of the PI3K/Akt signaling pathway in this model. The present work has the potential to yield significant insights into the relationship between HSOS and the gut microbiota, hence offering a promising avenue for the treatment of HSOS.

## 2 Materials and methods

### 2.1 Chemicals and reagents

Monocrotaline (MCT) was purchased from Aladdin Scientific Co. (Shanghai, China). Rivaroxaban was purchased from Bayer Pharma AG (Leverkusen, Germany). Primary antibodies, including phospho-PI3K (YP0224), PI3K (YM8045), and Akt (YM8463), were purchased from Immunoway Technology, Inc. (San Jose, United States). Additional primary antibodies, including phospho-AKT (66444-1-Ig), Cyclin D2 (10934-1-AP), and P27KIP1 (25614-1-AP), were purchased from Proteintech Group, Inc. (Rosemont, United States). Other primary antibodies, including anti-IGF1 (bs-4588R), were purchased from Bioss Biotechnology Co., Ltd. (Beijing, China). Anti-GAPDH (BL072A) was purchased from Beijing Labgic Technology Co., Ltd. (Beijing, China). Finally, Proteintech Group, Inc. (Rosemont, Uuited States) supplied goat anti-rabbit IgG (SA00001-2). Shanghai Shenggong Bioengineering Co., Ltd. synthesized all the experiment-related primers.

### 2.2 Animals

Forty male C57BL/6 J mice, 10–12 weeks old and weighing 25–30 g, were purchased from SBF Biotechnology Co. (Beijing, China). The mice were housed in specific-pathogen-free (SPF) facilities at a temperature of 24 ± 2°C, a relative humidity of 50% ± 10%, and a 12-h/12-h day/night cycle. All the mice were provided with food and water ad libitum. The mice were allowed to adapt to the environment for 1 week before the experiment. This study was conducted in strict accordance with the guidelines of the National Institutes of Health (NIH) and was approved by the Animal Research Committee of China Medical University (TZ2020052).

### 2.3 Experimental design

After acclimatization for 1 week, the mice were administered a monocrotaline (MCT) + PBS solution (30 mg/mL, 450 mg/kg) by oral gavage at 0 h to induce MCT-HSOS *in vivo*, and the mice’s weights were recorded daily.

**Figure 1:**
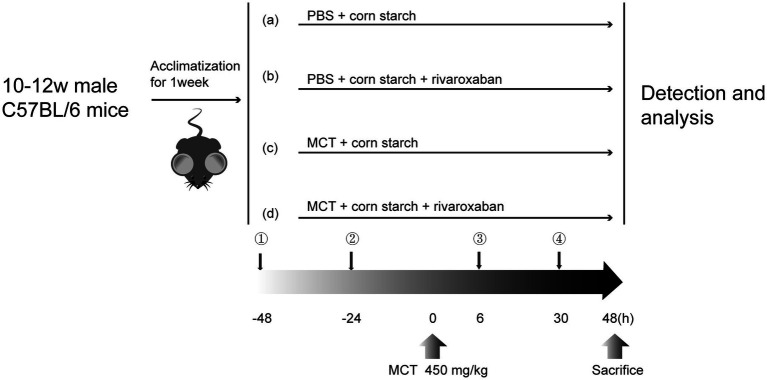
Flowchart of the experimental design. (a) Control group; (b) RIV group; (c) MCT group; (d) MCT + RIV group.

The mice were divided randomly into four groups (Figure 1, *n* = 10 per group): A, the control group: the mice were given PBS at 0 h and corn starch solution (6.18 mg/kg mice) at −48, −24, 6, and 30 h without monocrotaline and rivaroxaban by oral gavage; B, the RIV group: the mice were given PBS and rivaroxaban (RIV, 6.18 mg/kg mice) + corn starch solution (1.5 mg/mL) without monocrotaline by oral gavage; C, the MCT group: the mice were given monocrotaline + PBS and corn starch solution without rivaroxaban by oral gavage; D, the MCT + RIV group: the mice were given monocrotaline + PBS and rivaroxaban + corn starch solution by oral gavage. At 48 h after MCT, the mice were anesthetized with pentobarbital sodium (40 mg/kg) via intraperitoneal injection. Next, the serum, liver, small intestine, and small intestine contents were collected from the mice. Part of the left lobe (1 cm^3^) was fixed in 4% paraformaldehyde for histology. The remaining samples were snap-frozen in liquid nitrogen and stored at −80°C.

### 2.4 Antibiotic cocktail (ABX) administration

**Figure 2:**
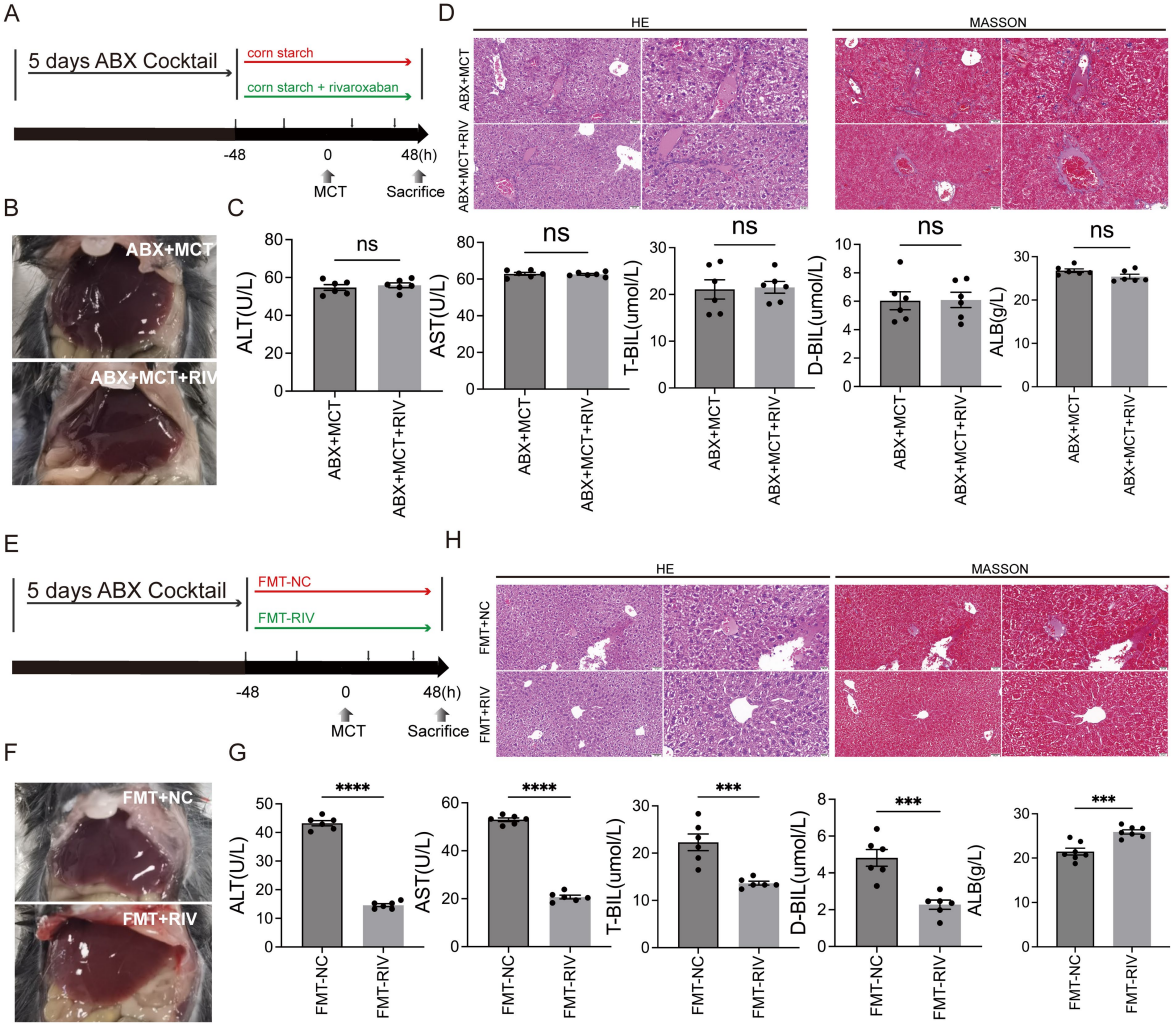
The gut microbiota mediates the protective effect of rivaroxaban against MCT-induced HSOS. **(A)** Schematic diagram of the ABX experimental protocol. **(B)** Hepatic morphology of the mice in the ABX + MCT and ABX + MCT + RIV groups (*n* = 6). **(C)** Serum liver function biochemical analysis of liver function in the ABX + MCT and ABX + MCT + RIV groups. Statistical analysis was performed using an unpaired *t*-test. The data are presented as the means ± SEMs (*n* = 6). **(D)** HE and Masson staining of liver tissues from the ABX + MCT and ABX + MCT + RIV groups. Images were captured at 200 × magnification (scale bar = 50 μm) and 400 × magnification (scale bar = 20 μm) (*n* = 6). **(E)** Schematic diagram of the FMT experimental protocol. **(F)** Hepatic morphology of the mice in the FMT-NC and FMT-RIV groups (*n* = 6 per group). **(G)** Serum liver function biochemical analysis of liver function in the FMT-NC and FMT-RIV groups. Statistical analysis was performed using an unpaired *t*-test. The data are presented as the means ± SEMs (*n* = 6). ***p < 0.001, ****p < 0.0001. **(H)** HE and Masson staining of liver tissues from the FMT-NC and FMT-RIV groups. Images were captured at 200 × magnification (scale bar = 50 μm) and 400 × magnification (scale bar = 20 μm) (*n* = 6).

In accordance with the experimental protocol, 12 mice were randomly assigned to either the ABX + MCT group or the ABX + MCT + RIV group (*n* = 6 per group). To eliminate the gut microbiota, all the mice were orally administered an antibiotic cocktail once daily for five consecutive days. The cocktail consisted of vancomycin, 100 mg/kg (Y25829, Shanghai YuanYe Biotechnology Co., Ltd., Shanghai, China); neomycin sulfate, 200 mg/kg (S17028, Shanghai YuanYe Biotechnology Co., Ltd., Shanghai, China); metronidazole, 200 mg/kg (S17079, Shanghai YuanYe Biotechnology Co., Ltd., Shanghai, China); and ampicillin, 200 mg/kg (S17018, Shanghai YuanYe Biotechnology Co., Ltd., Shanghai, China). Following ABX administration, the mice in the ABX + MCT group received oral monocrotaline and corn starch solution without rivaroxaban. In contrast, the mice in the ABX + MCT + RIV group were orally administered an equivalent volume of monocrotaline, rivaroxaban, or corn starch solution, as described above (Figure 2a). All mice were housed in cages with metal grid flooring, which prevented them from accessing feces that had fallen to the bottom of the cage, and mice from each experimental group were kept in separate cages.

### 2.5 Fecal microbiota transplantation

To prepare the bacterial suspension, 120 mg of freshly excreted fecal samples were immediately collected from mice that had been treated with either corn starch solution or rivaroxaban. The feces were dissolved in 1.5 mL of sterile PBS containing 15% glycerol, thoroughly vortexed, and then centrifuged at 1,000 × g for 3 min at 4°C. The supernatant was collected to remove insoluble debris and stored at −80°C for future use. Recipient mice were pretreated with an antibiotic cocktail via oral gavage once daily for 5 consecutive days to deplete the gut microbiota. The antibiotic cocktail consisted of vancomycin (100 mg/kg), neomycin sulfate (200 mg/kg), metronidazole (200 mg/kg), and ampicillin (200 mg/kg). After antibiotic treatment, 12 recipient mice were randomly assigned to either the FMT-NC or FMT-RIV group (*n* = 6 per group) and were administered 10 mL/kg of the corresponding fecal suspension and monocrotaline (MCT) by oral gavage, as described above (Figure 2e). All mice were housed in cages with metal grid flooring, which prevented them from accessing feces that had fallen to the bottom of the cage, and mice from each experimental group were kept in separate cages.

### 2.6 Serum biochemical testing

The alanine aminotransferase (ALT), aspartate aminotransferase (AST), total bilirubin (T-BIL), direct bilirubin (D-BIL), and albumin (ALB) levels in the serum samples were evaluated using an assay kit (Nanjing Jiancheng Bioengineering Institute, Nanjing, China) according to the manufacturer’s instructions.

### 2.7 Liver histology

Liver tissue was fixed in 4% paraformaldehyde for at least 24 h. Then, the tissues were embedded in paraffin and sectioned into 5-μm slices. The slides were stained with hematoxylin (H8070, Solarbio, Beijing, China)-eosin stain (A600190, Sangong, Shanghai, China) and a Trichrome (Masson) Stain Kit (G1340, Solarbio, Beijing, China) according to the manufacturer’s instructions. We evaluated the *severity* of HSOS by examining histological changes in central venule endothelial cell injury, coagulative necrosis of liver cells, subendothelial hemorrhage of the central vein, and hepatic sinusoidal hemorrhage. The items were rated on a 4-point scale and summed to obtain a total score. Table 1 lists the specific scoring rules.

**Table 1:** MCT-HSOS hepatic histology injury scores (DeLeve et al., 1999).

Central venule endothelial cell injury	Coagulative necrosis of liver cells	Subendothelial hemorrhage of the central vein	Hepatic sinusoidal hemorrhage
0, none	0, none	0, none	0, none
1, mild (Local involvement, but not all inner walls of endothelial vessels)	1, mild (Some but not all lobules are involved, or most lobules are not involved between lobules)	1, mild (Some but a few CVs are involved, or most CVs do not involve the interlobular area)	1, mild (The blood sinus in the central area of the lobule is involved, or most of the central area of the lobule is involved, but the interlobular area is not involved)
2, moderate (The whole inner wall of a few vessels is involved)	2, moderate (The lobular center is involved, and a few interlobular areas are involved)	2, moderate (Most CVs are involved, and some but a few CVs are involved in interlobular areas);	2, moderate (The central area of lobules is mostly involved, and a few lobules are involved in interlobular areas)
3, severity (The whole inner layer of the vessel is affected by most vessels)	3, severity (Most lobular centers are involved, and most interlobular areas are involved).	3, severity (Most CVs are involved, and most CVs are involved in interlobular areas).	3, severity (The central area of the lobule is mostly affected, and most of the lobules are involved in the interlobular area).

### 2.8 16S rDNA sequencing and analysis

The intestinal contents of 22 mice were collected for 16S rDNA sequence analysis. *There were* 5–6 *samples in* each *group.* Total genomic DNA was extracted from the samples using an Omega soil DNA kit (M5635-02) (Omega Bio-tek, Norcross, GA, United States) according to the manufacturer’s instructions. The quantity of DNA was determined with a NanoDrop NC2000 spectrophotometer (Thermo Fisher Scientific, Waltham, MA, United States). The quality of the DNA was determined via agarose gel electrophoresis. The V3-V4 region of the bacterial 16S rRNA gene was amplified with the forward primer 338F and reverse primer 806R. The PCR amplification product was purified using Vazyme VAHTSTM DNA Clean beads (Vazyme, Nanjing, China) and quantified with a Quant-iT PicoGreen dsDNA assay kit (Invitrogen, Carlsbad, CA, United States). Pair-end 2×250 bp sequencing was performed at Shanghai Personal Biotechnology Co. Ltd. (Shanghai, China) via the Illumina NovaSeq platform and NovaSeq 6,000 SP kit (500 cycles). Each OTU representative sequence was annotated via QIIME2 software with the Greengenes database and the QIIME2 classify-sky learning algorithm. The sequencing data were processed via QIIME2 version 2019.4, R software and STAMP software^1^ (Parks et al., 2014).

### 2.9 Small intestine transcriptome sequencing and analysis

After the mice were sacrificed, the small intestine was collected and snap-frozen in liquid nitrogen to prevent RNA degradation. We sent the samples on dry ice to Shanghai Personal Biotechnology Co., Ltd. (Shanghai, China). Total RNA extraction and quality control of the small intestine, cDNA library creation and quality control, sequencing on the Illumina NovaSeq platform, and subsequent transcriptome data analysis were subsequently performed (significantly differentially expressed genes (DEGs) with expression difference multiplicity |log_2_FoldChange| > 1, significance *p*-value<0.05). Differentially expressed genes (DEGs) were annotated via the Gene Ontology (GO) database and the Kyoto Encyclopedia of Genes and Genomes (KEGG). Sequencing and bioinformatics analysis were performed in strict accordance with the company’s standard operating procedures.^2^

### 2.10 Real-time qPCR

IGF1, PI3K, AKT1, P27, and CCND2 in liver tissue were measured via real-time qPCR. Total RNA was extracted from 50 mg of liver tissue via RNAiso Plus (9,108, TAKARA, Shiga, Japan). The concentration and purity of the RNA were determined via a Nanodrop 2000 (Thermo Fisher, United States). The RNA was reverse-transcribed into cDNA via the All-In-One 5 × RT MasterMix Kit (G592, Abm, BC, CANADA). Then, real-time qPCR was performed using BlasTaqTM 2X qPCR MasterMix (G891, Abm, BC, Canada) and a LightCycler 480 II System PCR instrument (Roche, Switzerland) according to the manufacturer’s protocols. GAPDH was used as an internal reference. The 2^–ΔΔCt^ method for relative quantification was used to compare the differences in mRNA expression. The primers used are listed in Table 2.

**Table 2:** Real-time qPCR primer sequences.

Gene	Forward (5’ to 3’)	Reverse (5’ to 3’)
IGF1	*GAG GGG CTT TTA CTT CAA CAA G*	*TAC ATC TCC AGT CTC CTC AGA T*
PI3K	*AAA CAA AGC GGA GAA CCT ATT G*	*TAA TGA CGC AAT GCT TGA CTT C*
AKT1	*TGC ACA AAC GAG GGG AAT ATA T*	*CGT TCC TTG TAG CCA ATA AAG G*
P27	*TTT AAT TGG GTC TCA GGC AAA C*	*CCC TTT TGT TTT GCG AAG AAG A*
CCND2	*GAC CGT TTC TTG GCT GGA GT*	*CAC AGC TTT TCC GCA GTC AG*
GAPDH	*GTT GTC TCC TGC GAC TTC A*	*TGG TCC AGG GTT TCT TAC TCC*

### 2.11 Western blot analysis

RIPA lysis buffer (P0013B, Beyotime Biotechnology, Shanghai, China) containing PMSF (ST505, Beyotime Biotechnology, Shanghai, China), peptidase inhibitor (P001, New Cell & Molecular Biotech Co., Ltd., Suzhou, China), and phosphatase inhibitor (P003, New Cell & Molecular Biotech Co., Ltd., Suzhou, China) was applied to the tissue. The lysates were incubated at low temperatures for 25 min, and after centrifugation at 4°C and 12,000 rpm·min^−1^ for 8 min, the supernatant was aspirated into a centrifuge tube. The protein concentration of the supernatant was quantified via a BCA assay kit (WB6501, New Cell & Molecular Biotech Co., Ltd., Suzhou, China). Protein samples (45 μg) were electrophoretically separated on sodium dodecyl sulfate-polyacrylamide gels and then transferred to PVDF membranes (10,600,023, Cytiva, Buckinghamshire, United Kingdom), which were incubated for 1.5 h at room temperature. Primary antibody incubation was performed overnight. The following day, the WB strips were incubated with the corresponding secondary antibody for 90 min. The membranes were treated with an enhanced chemiluminescence (ECL) detection reagent (180--501, Tanon, Shanghai, China). The relative band intensity was determined via ImageJ software (National Institutes of Health, Bethesda, MD, United States).

### 2.12 Spearman correlation coefficient analysis

Correlations between the gut microbiota and serum biochemical parameters, as well as gene expression in small intestinal tissues and liver tissues, were calculated and analyzed using Spearman’s rank correlation coefficients. This analysis was performed via the GenesCloud tool.^3^

### 2.13 Statistical analysis

The data are expressed as the means with SEMs and means with 95% CIs. Unpaired *t* tests and Welch’s *t* tests were used to compare two groups. One-way ANOVA and Tukey’s test were used to compare three or more groups. *p* < 0.05 was considered statistically significant. All experimental data were statistically analyzed with GraphPad Prism 8.0 software (GraphPad Software, La Jolla, CA, United States) and STAMP software.

## 3 Results

### 3.1 Serum liver function biochemical analysis and liver histopathology

**Figure 3:**
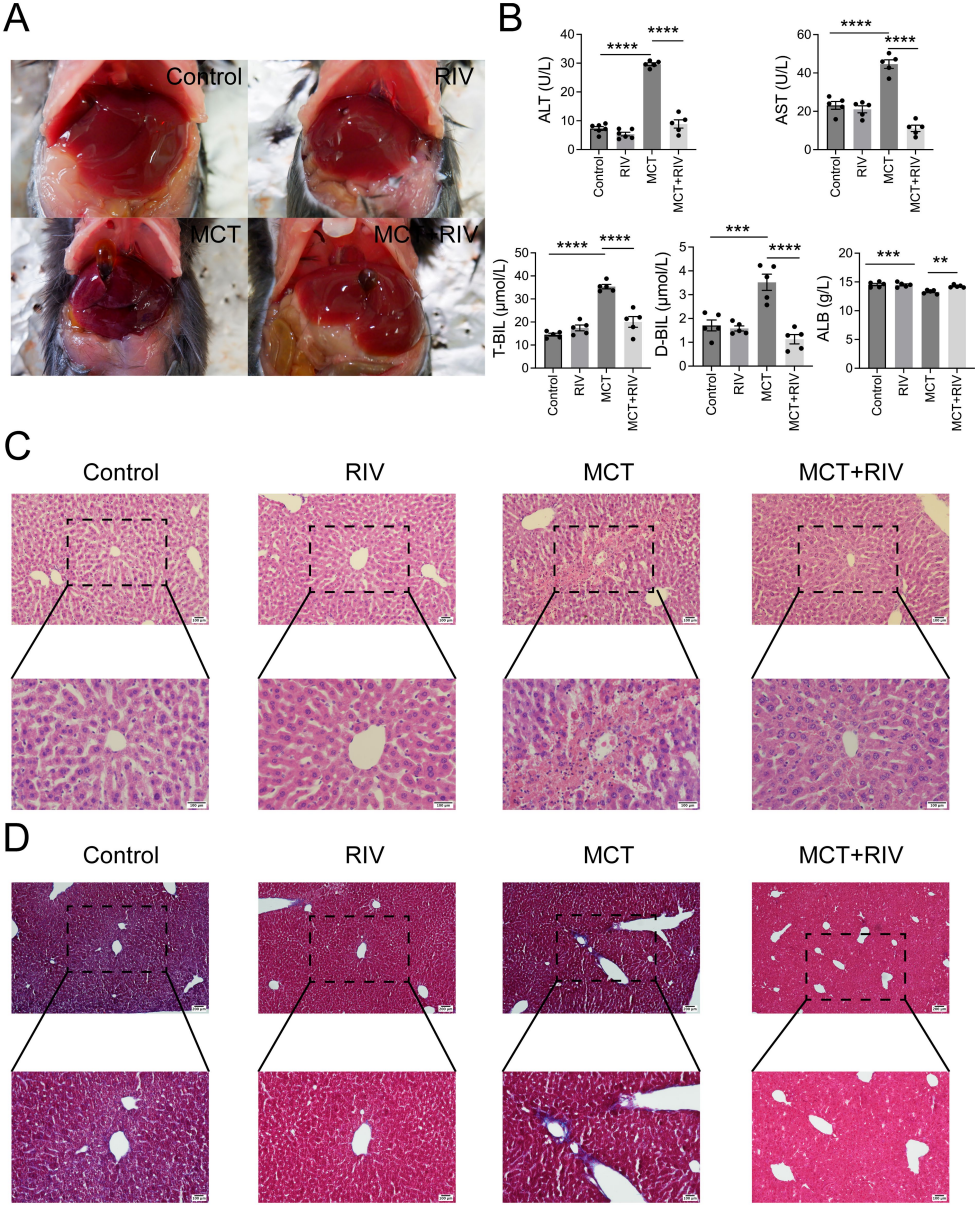
Rivaroxaban ameliorated MCT-induced HSOS in mice. **(A)** Hepatic morphology (*n* = 5). **(B)** Serum liver function biochemical analysis. Statistical analysis was performed via ordinary one-way ANOVA. The data are presented as the means ± SEMs (*n* = 5). **p < 0.005, ***p < 0.001, ****p < 0.0001. **(C)** HE staining of liver tissues. Representative images from each group are shown (upper panel: 200 × magnification, scale bar = 100 μm; lower panel: 400 × magnification, scale bar = 100 μm) (*n* = 10). **(D)** Masson staining of liver tissues. Representative images from each group are shown (upper panel: 100 × magnification, scale bar = 200 μm; lower panel: 200 × magnification, scale bar = 100 μm) (*n* = 100). *p < 0.05..

To evaluate the effect of rivaroxaban on MCT-HSOS in mice, we examined serum biochemistry and liver histology. At 48 h after MCT administration, the livers of the MCT group were dark red and rough. Compared with those in the MCT group, the livers in the MCT + RIV group were ruddy and smooth (Figure 3a).

The results of the serum biochemistry data are shown in Figure 3b. The serum ALT, AST, T-BIL, and D-BIL levels in the MCT + RIV group were significantly lower than those in the MCT group (*p* < 0.0001). In addition, the ALB level in the MCT + RIV group was significantly greater than that in the MCT group (*p* < 0.005).

The HE staining results (Figure 3c) revealed that the liver structure in the MCT group was disrupted. In the MCT group, more severe central venule endothelial cell injury, coagulative necrosis of liver cells, subendothelial hemorrhage of the central vein, and hepatic sinusoidal hemorrhage were observed. Compared with those in the MCT group, these pathological manifestations in the MCT + RIV group were alleviated. The histological score of the MCT + RIV group was significantly lower than that of the MCT group (*p* < 0.05) (Table 3).

**Table 3:** HSOS scores of liver tissues 48 h after MCT administration.

Scores (range)	MCT	MCT + RIV
Central venule endothelial cell injury (0–3)	2.5 ± 0.2	1.7 ± 0.3*
Coagulative necrosis of liver cells (0–3)	1.6 ± 0.2	0.8 ± 0.2*
Subendothelial hemorrhage of the central vein (0–3)	1.6 ± 0.2	0.7 ± 0.3*
Hepatic sinusoidal hemorrhage (0–3)	1.9 ± 0.2	1.6 ± 0.2
Total HSOS score (0–9)	6 ± 0.5	4.2 ± 0.5*

*p < 0.05.

The total HSOS score was calculated as the sum of individual scores for central venule endothelial cell injury, subendothelial or sinusoidal hemorrhage (highest bleeding score), and coagulative necrosis in each mouse. The data are presented as the mean ± SEM (*n* = 10). Statistical analysis was performed using an unpaired *t*-test. *p < 0.05 compared with the MCT group.

We also performed Masson staining on liver tissue to observe fibrogenesis around liver cells (Figure 3d). MCT promoted fibrosis in the hepatic sinusoids and portal areas of a few mice. Compared with that in the MCT group, the amount of fiber tissue in the MCT + RIV group was lower.

### 3.2 Rivaroxaban regulated the gut microbiota of MCT-HSOS mice

**Figure 4:**
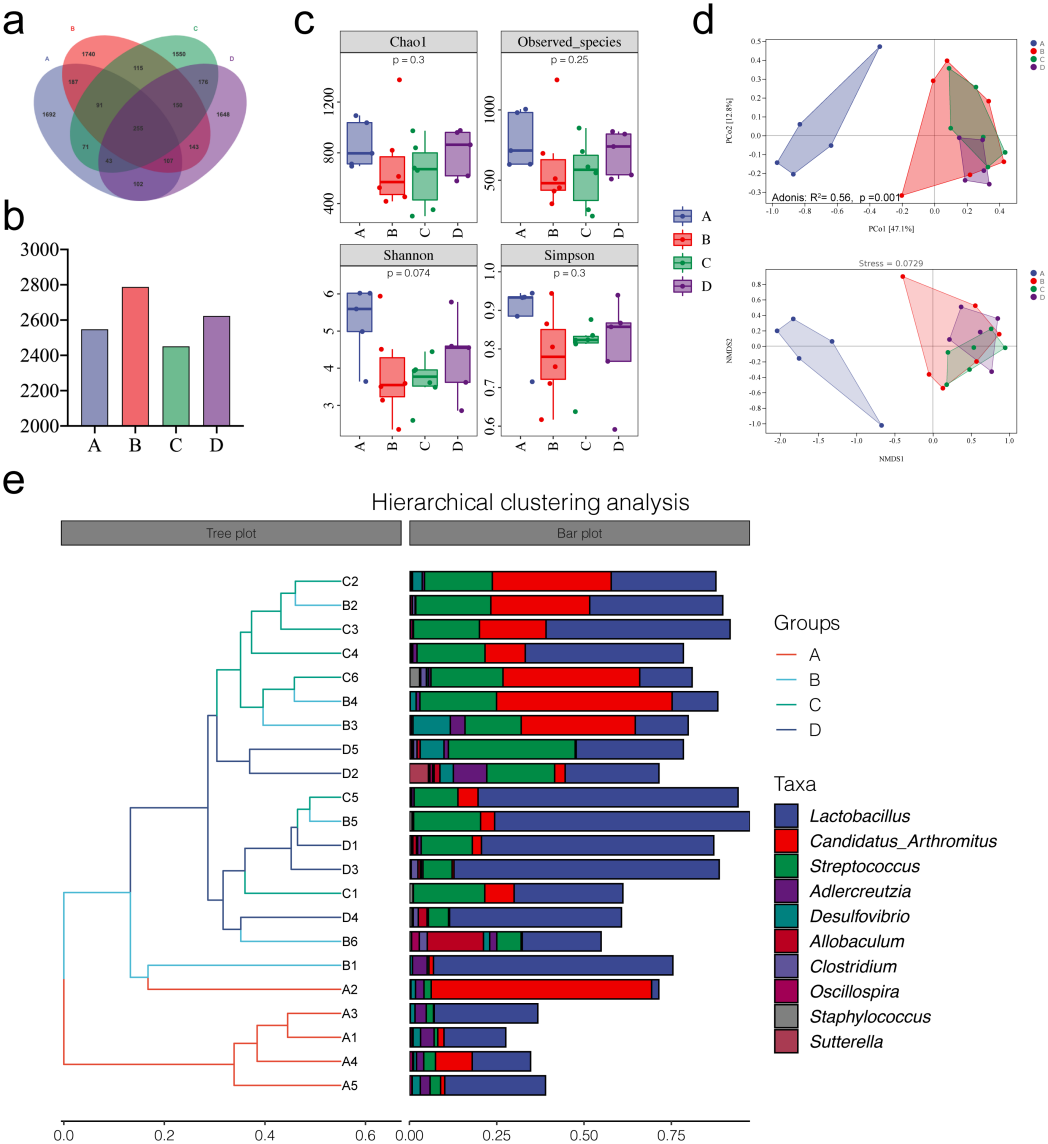
Microbial diversity analysis of the different groups. **(a)** OTU Venn diagram. **(b)** Total OTUs in each sample group. **(c)** Alpha diversity analysis of each group. **(d)** PCoA, Adonis, and NMDS analyses. **(e)** Hierarchical clustering analysis. (A, Control group, *n* = 5; B, RIV group, *n* = 6; C, MCT group, *n* = 6; D, MCT + RIV group, *n* = 5).

We performed 16S rDNA sequencing of small intestinal contents from mice to investigate whether rivaroxaban affects HSOS by modulating the gut microbiota. The OTUs were divided according to 100% identity to understand the bacterial species. As shown in Figures 4a,b, the control group and MCT group shared 460 OTUs. The control group and MCT + RIV group shared 507 OTUs. The number of shared OTUs of the latter was greater than that of the former.

Alpha diversity indices include the Chao1 estimator, observed indices, and the Shannon and Simpson indices. We evaluated richness using the Chao1 estimator and observed specificity indices, as well as diversity using the Shannon and Simpson indices. As shown in Figure 4c, there was no significant difference between the groups (*p* > 0.05); however, MCT had the potential to reduce alpha diversity indices. Compared with those in the MCT group, each index in the MCT + RIV group tended to increase. These findings suggest that rivaroxaban has the potential to improve the richness and diversity of the intestinal microbiota in the MCT-HSOS model mice. In the beta diversity analysis results, the results of principal coordinate analysis (PCoA), adonis analysis, nonmetric multidimensional scaling analysis (NMDS), and hierarchical clustering analysis, which are all based on the weighted UniFrac distance, revealed significant differences in the structure of the gut microbiota between groups (Adonis: *R*^2^ = 0.56, *p* = 0.001) (Figures 4d,e).

**Figure 5:**
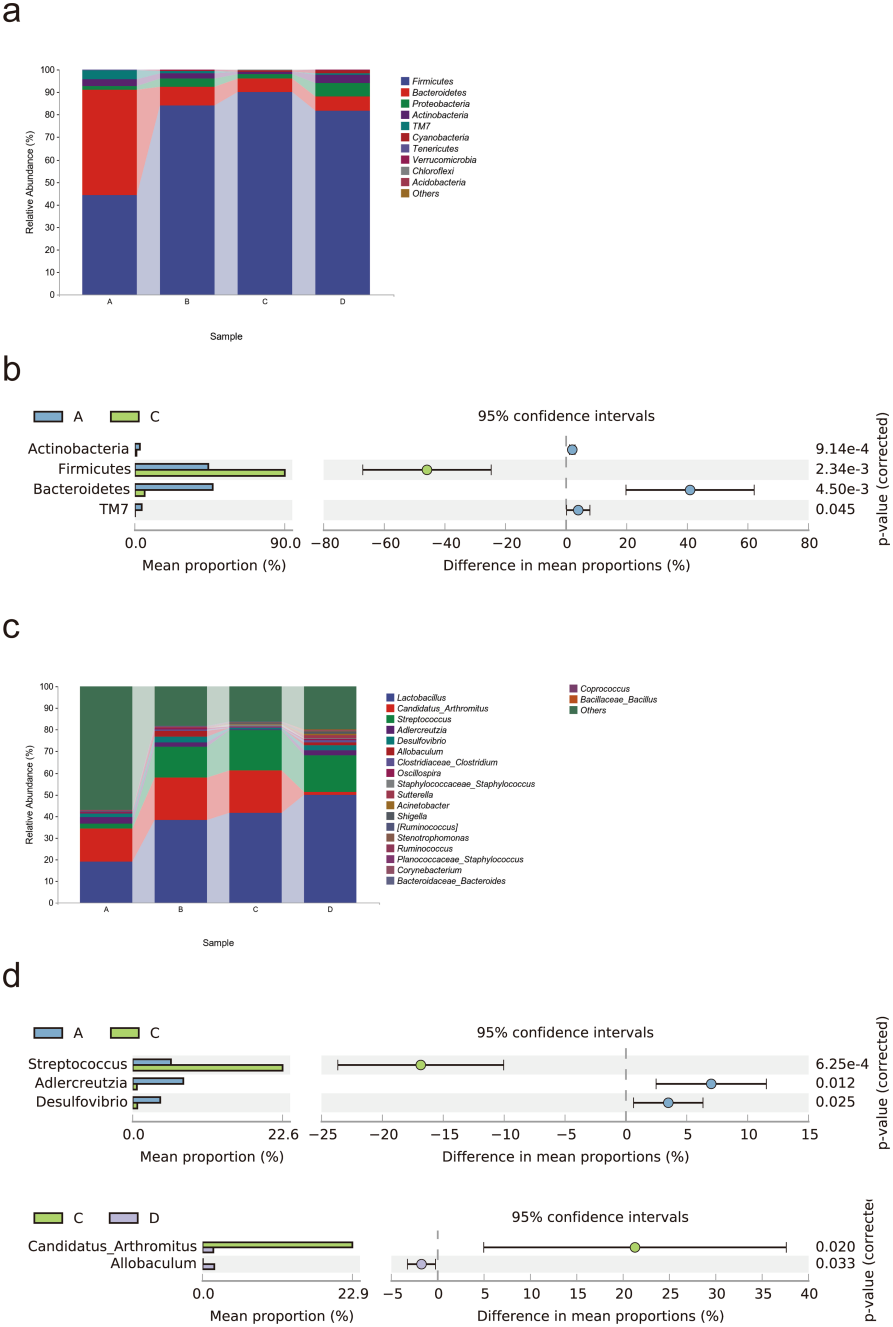
Rivaroxaban modulates the gut microbial composition in MCT-HSOS model mice. **(a)** Taxonomic composition analysis at the phylum level. **(b)** Differentially abundant bacterial phyla between groups (*p* < 0.05, computed by STAMP). **(c)** Taxonomic composition analysis at the genus level. **(d)** Differentially abundant bacteria between groups (*p* < 0.05, computed by STAMP). Statistical analysis was performed using a two-sided Welch’s *t*-test. The data are expressed as the means with 95% CIs. (A. Control group, *n* = 5; B, RIV group, *n* = 6; C, MCT group, *n* = 6; D, MCT + RIV group, *n* = 5).

We studied the microbial composition of each sample at the phylum and genus levels through species composition analysis. At the phylum level (Figures 5a,b), the relative abundances of *Actinobacteria*, *Bacteroidetes*, and *TM7* were lower, and the relative abundance of *Firmicutes* was greater in the MCT group than in the control group (*p* < 0.05).

At the genus level (Figures 5c,d), the relative abundances of *Adlercreutzia* and *Desulfovibrio* (*p* < 0.05) and the relative abundance of *Streptococcus* (*p* < 0.05) were lower in the MCT group than in the control group. Compared with the MCT group, the MCT + RIV group presented a decreased relative abundance of *Candidatus Arthromitus* (*p* < 0.05) and an increased relative abundance of *Allobaculum* (*p* < 0.05).

**Figure 6:**
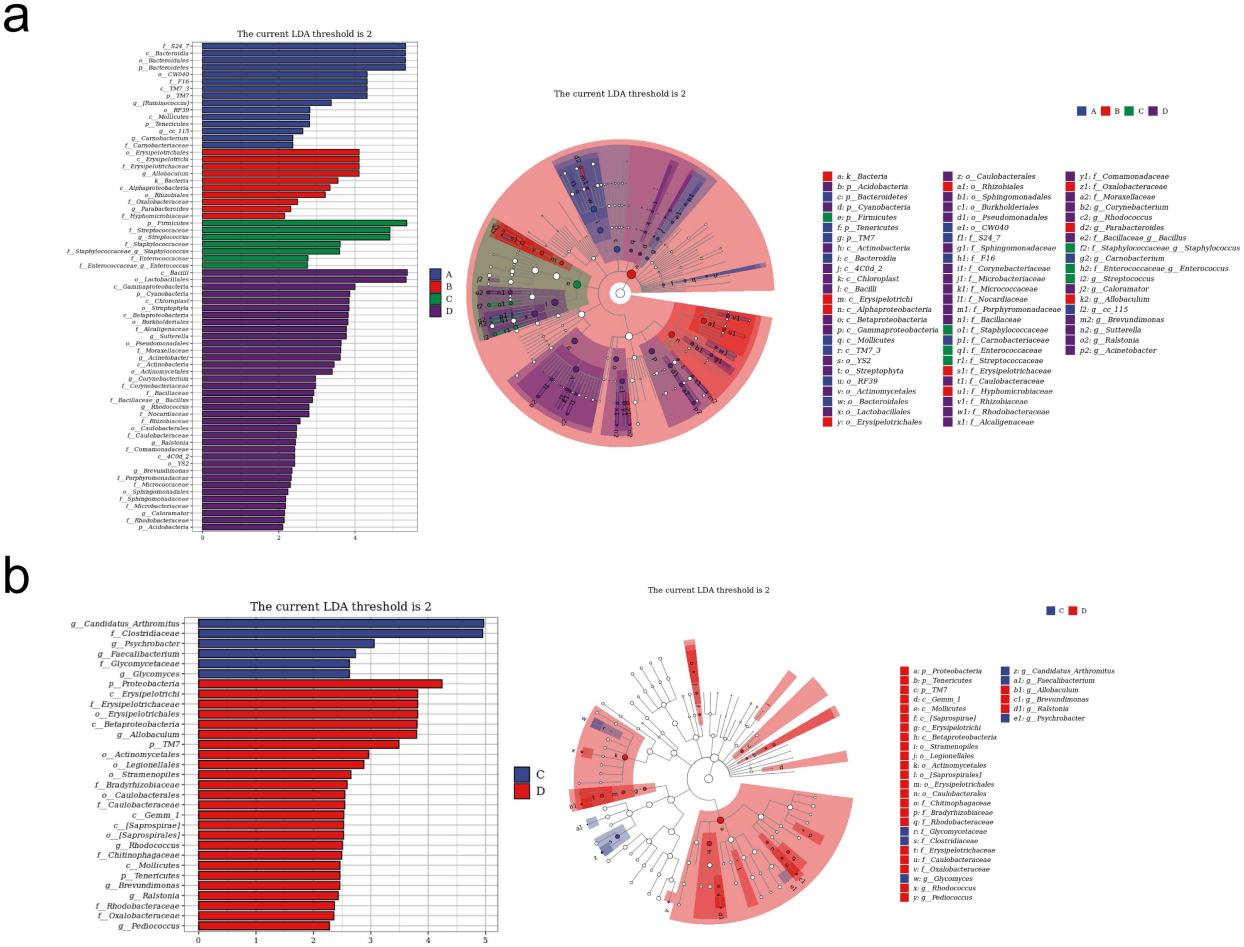
LEfSe analysis of the gut microbiota in different groups. **(a)** LEfSe analysis histogram and cladogram for all four groups (A, B, C, and D). **(b)** LEfSe analysis histogram and cladogram for groups C and D. (A) Control group (*n* = 5); (B) RIV group (*n* = 6); (C) MCT group (*n* = 6); (D) MCT + RIV group (*n* = 5).

LEfSe analysis was used to find taxa with differences in abundance among groups. We conducted LEfSe analysis on the control group, RIV group, MCT group, and MCT + RIV group based on an LDA score of >2.0 and a *p*-value of <0.05 (Figure 6a). Compared with those in the other three groups, the relative abundances of *Bacteroidetes* and *TM7* in the control group increased, while the relative abundances of Firmicutes, Streptococcus, and Staphylococcus in the MCT group increased, and the relative abundance of Allobaculum in the RIV group increased (LDA score > 2.0, *p* < 0.05). To further explore the effects of rivaroxaban on MCT-HSOS in mice, we conducted LEfSe analysis for the MCT group and the MCT + RIV group on the basis of an LDA score>2.0, *p* < 0.05 standard (Figure 6b). Compared with each other, *Candidatus Arthromitus* in the MCT group increased, and *Allobaculum* and *Pediococcus* in the MCT + RIV group increased (LDA score >2.0, *p* < 0.05).

### 3.3 Results of transcriptomic analysis of mouse small intestine tissue

To further investigate the molecular mechanism of rivaroxaban’s effect on HSOS, we performed transcriptome sequencing and analysis of small intestinal tissues from mice.

**Figure 7:**
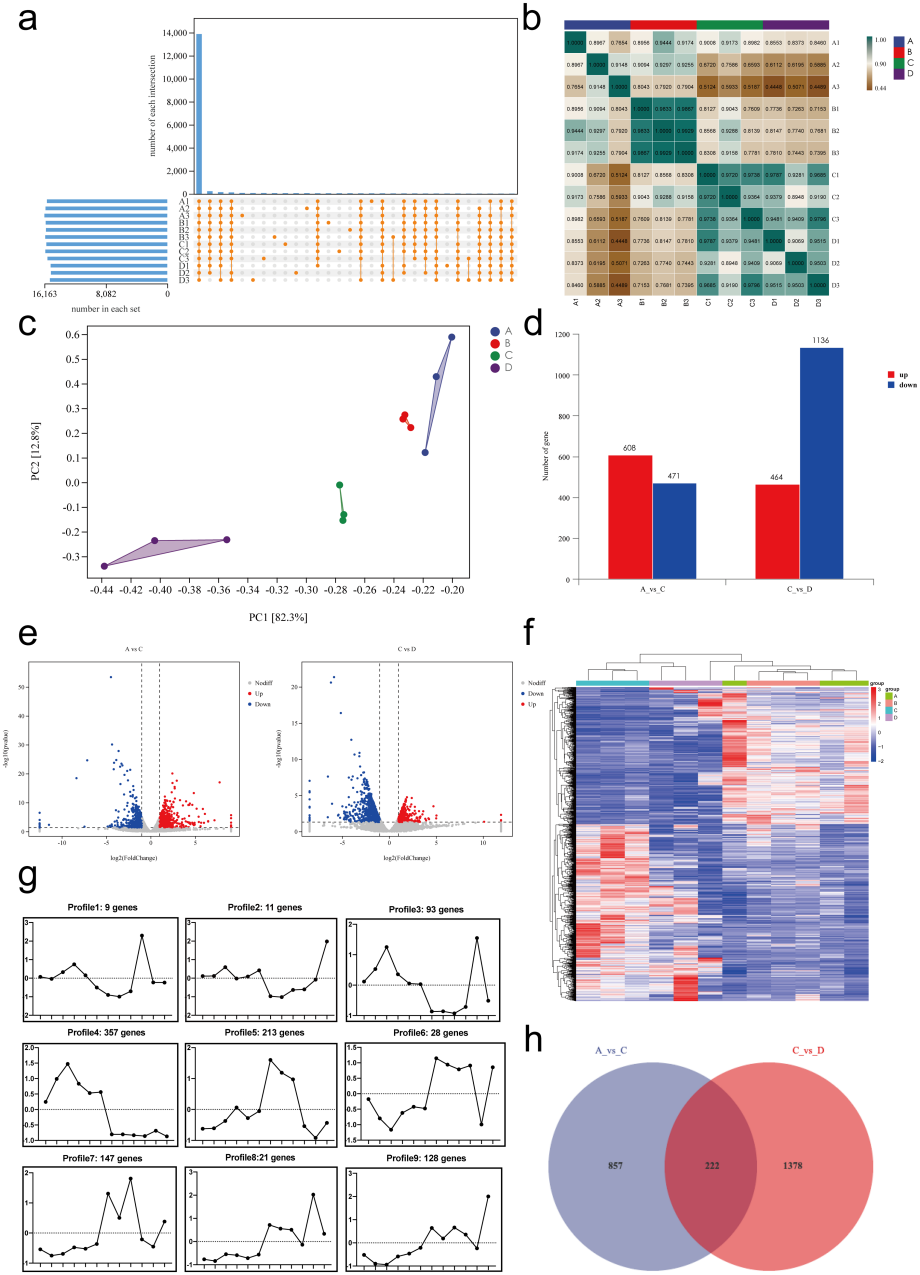
Transcriptomic analysis of mRNA expression and differential gene expression in small intestinal tissues. **(a)** Gene expression analysis. **(b)** Sample correlation analysis. **(c)** Principal component analysis (PCA). **(d)** Summary of differentially expressed genes. **(e)** Volcano plots of differentially expressed genes: A vs. C and C vs. D. **(f)** Cluster analysis: genes are represented horizontally, with red indicating high expression and green indicating low expression. **(g)** Trend analysis: the x-axis represents samples arranged sequentially from A to D, and the y-axis represents the average expression level of all genes in each sample. The number of genes corresponding to each cluster is shown in the header. **(h)** Venn diagram of DEGs between A vs. C and C vs. D. A, Control group, *n* = 5; B, RIV group, *n* = 6; C, MCT group, *n* = 6; D, MCT + RIV group, *n* = 5.

We normalized the sequencing data with fragments per kilobase per million fragments (FPKM) values. First, we determined the expression of genes, and the results are shown in Figure 7a: 13,875 genes were differentially expressed genes (DEGs) between samples, and only a few genes were uniquely expressed genes.

Next, we performed correlation analysis and principal component analysis (PCA) to assess the correlation between gene expression levels in different samples. The results are shown in Figure 7b, and the correlation coefficient for each sample within the group was greater than 0.76. PCA (Figure 7c) revealed small distances within each group and large distances between groups, indicating relative independence. PC1 explained 82.3% of the variance, and PC2 explained 12.8% of the variance. Both analyses indicated that the expression levels of genes were highly similar within groups and slightly different between groups.

The primary aim of transcriptomics is to identify significant DEGs, which are defined as those for which |log_2_FoldChange| > 1 and *p* < 0.05. Compared with the control group (A), the MCT group (C) had 608 significantly upregulated genes and 471 significantly downregulated genes. Compared with those in the MCT group (C), 464 genes in the MCT + RIV group (D) were significantly upregulated, and 1,136 genes were significantly downregulated. These significantly different genes may be target genes of rivaroxaban (Figures 7d,e).

Cluster analysis can classify genes with a similar trend among samples into a single cluster. These genes are usually functionally related. Therefore, cluster analysis and trend analysis could help us identify genes that conform to therapeutic trends. The analysis results (Figure 7f) revealed that the clustering distances of the samples in the control group (A) and the RIV group (B) were relatively close, while the samples in the MCT group (C) and the MCT + RIV group (D) were clustered separately. The trend analysis results for Clusters 1, 3, 5, and 7 are consistent with the trends observed in the previous experimental results. The genes encoding IGF1, TGFBR2, OSMR, FGFR4, EDA2R, and CCND2 (|log2FoldChange| > 1, *p* < 0.05) are significantly different from the genes corresponding to Cluster 5, and GDF15 and CCL24 (|log2FoldChange| > 1, *p* < 0.05) are genes corresponding to Cluster 7 (Figure 7g).

Finally, we performed a Venn diagram analysis of the DEGs. The Venn diagram (Figure 7h) revealed that 222 DEGs, including IGF1, TGFBR2, OSMR, FGFR4, EDA2R, CCND2, GDF15, and CCL24, overlapped between the comparison of the control group (A) vs. the MCT group (C) and the MCT group (C) vs. the MCT + RIV group (D).

### 3.4 Analysis of differential gene enrichment in the small intestine

**Figure 8:**
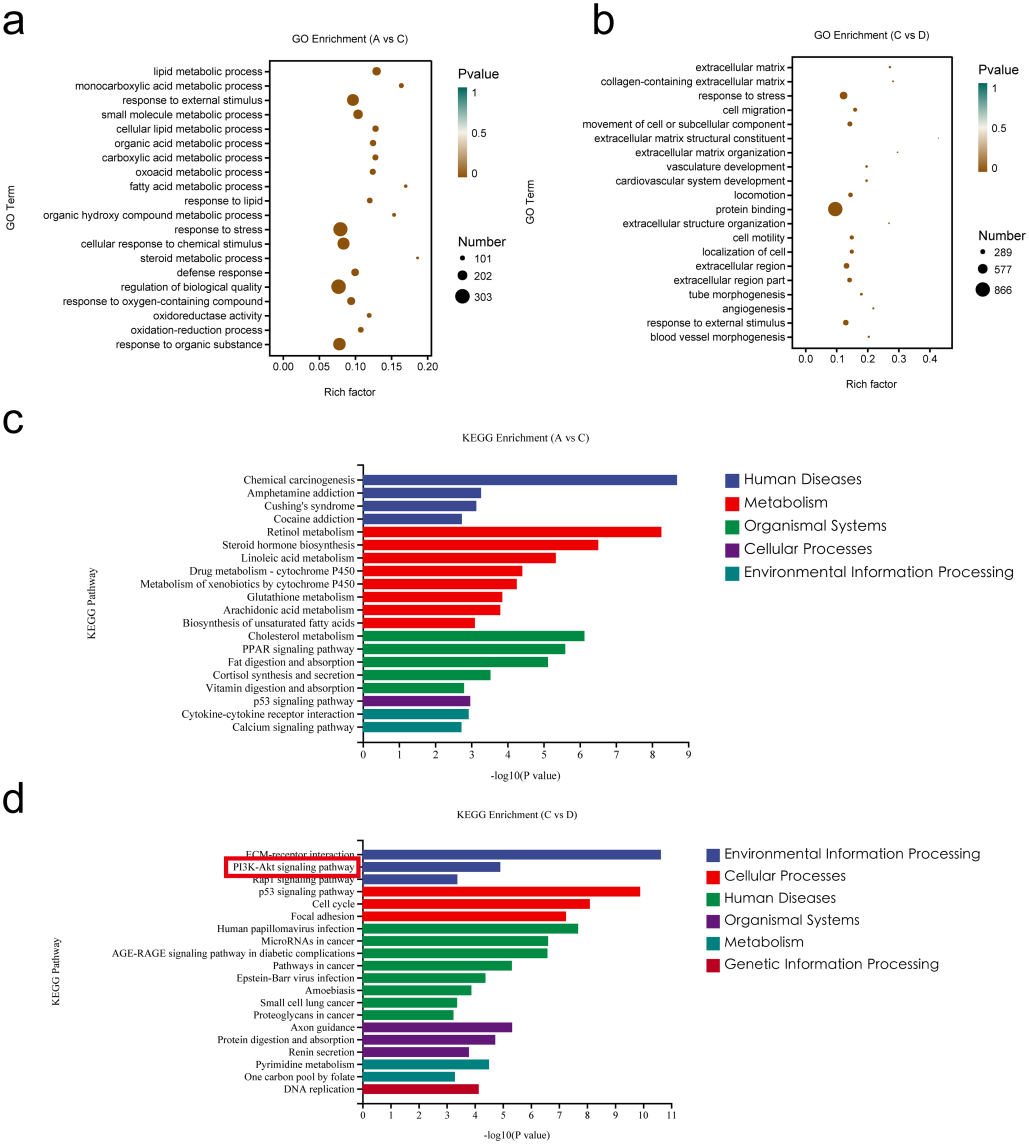
Enrichment analysis of differentially expressed genes in the small intestine. **(a)** GO enrichment analysis of DEGs between A and C. **(b)** GO enrichment analysis of DEGs between C and D. **(c)** KEGG pathway enrichment analysis of DEGs between A and C. **(d)** KEGG pathway enrichment analysis of DEGs between C and D. (A, Control group, *n* = 5; B, RIV group, *n* = 6; C, MCT group, *n* = 6; D, MCT + RIV group, *n* = 5).

To explore the functions and pathways associated with the DEGs *in vivo*, we performed GO and KEGG enrichment analyses on the DEGs. The GO terms (Figure 8a) that were significantly enriched (*p* < 0.05) for DEGs in the MCT group relative to the control group (A vs. C) included lipid metabolic processes and monocarboxylic acid metabolic processes. In addition, the significantly enriched terms in A vs. C included lipid metabolism, bile acid metabolism, the immune system, inflammatory reactions, cytokines, and vascular permeability. The following GO terms were significantly enriched (*p* < 0.05) in the MCT + RIV group relative to the MCT group (C vs. D) (Figure 8b): stress response, cell migration, and movement of cellular or subcellular components. In addition, the significantly enriched terms in the C vs. D comparison included the immune system, lipid metabolism, the inflammatory response, and cytokines. The terms related to the immune system included the antimicrobial humoral immune response mediated by antimicrobial peptides.

Among the enriched KEGG pathways (Figures 8c,d), the pathways that were significantly enriched and related to MCT-induced hepatic sinusoidal obstruction syndrome in mice included the MAPK signaling pathway, the PI3K-Akt signaling pathway, inflammatory mediator regulation of TRP channels, cytokine receptor interactions, the FoxO signaling pathway, and the TNF signaling pathway (*p* < 0.05).

The results revealed that the PI3K/Akt signaling pathway contained four significantly different genes and was the most enriched with significantly different genes among the above pathways. Therefore, the PI3K/Akt signaling pathway may be one of the pathways through which rivaroxaban prevents and treats the MCT-HSOS model mice.

### 3.5 Real-time qPCR and Western blot results for liver tissues

**Figure 9:**
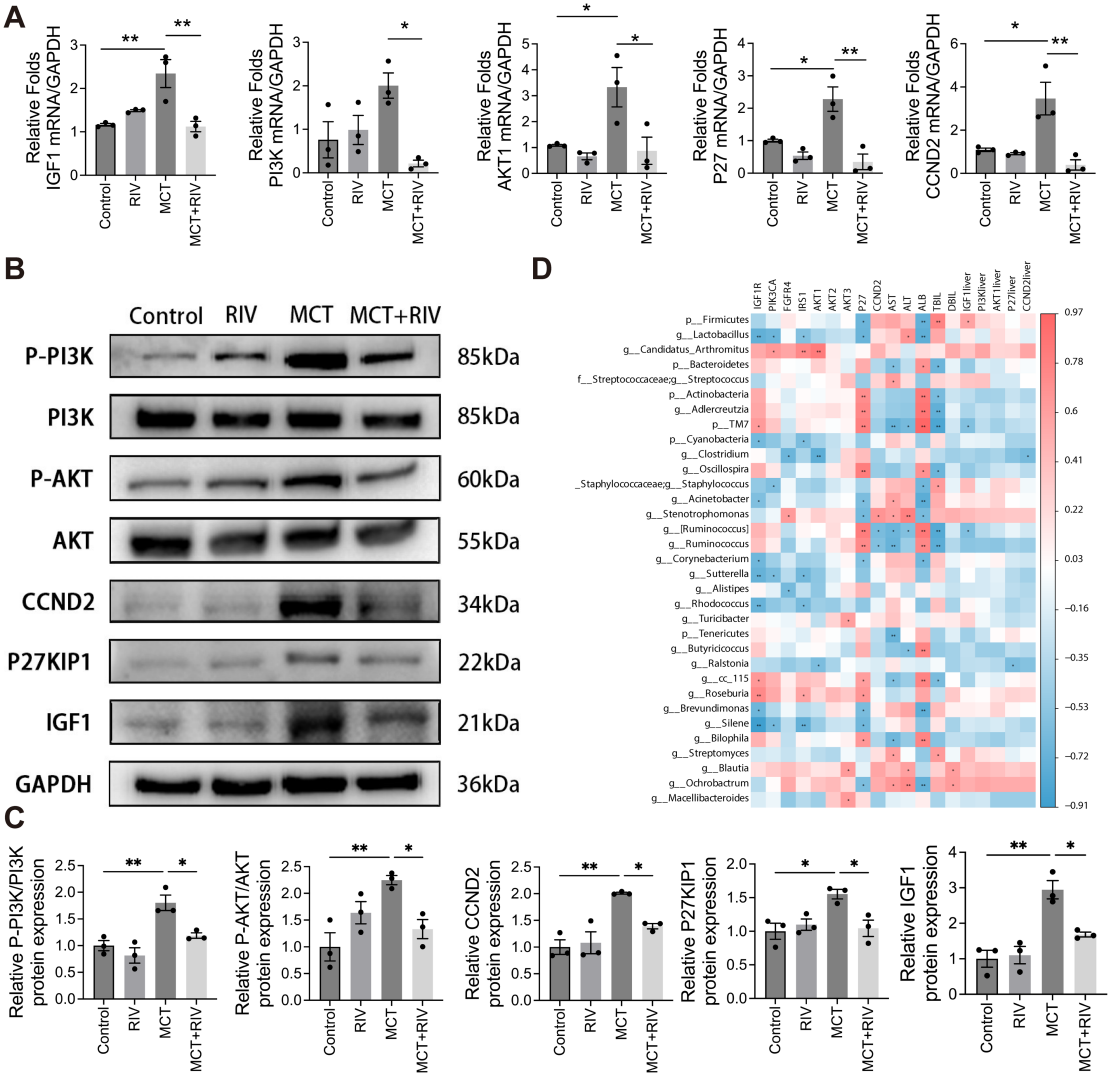
PI3K/Akt signaling pathway expression and correlation analysis. **(A)** Relative mRNA expression levels of PI3K/Akt signaling pathway-related genes in each group (*n* = 3). **(B)** Western blot analysis of hepatic protein levels of p-PI3K, PI3K, p-AKT, AKT, CCND2, P27KIP1, and IGF1. GAPDH was used as the loading control. **(C)** Quantitative assessment of p-PI3K/PI3K, p-AKT/AKT, CCND2, P27KIP1, and IGF1 protein levels (*n* = 3). **(D)** Heatmap depicting Spearman correlations among serum biochemical indices, small intestinal transcriptomics, liver tissue-associated genes, and the gut microbiota. The data are presented as the means ± SEMs. Statistical analysis was performed via ordinary one-way ANOVA. *p < 0.05, **p < 0.005, ***p < 0.001. All experiments were performed in triplicate, with a minimum of three replicates.

To investigate the expression of PI3K/Akt signaling pathway-related genes in mouse liver tissues, we performed real-time quantitative PCR (qPCR) and Western blot analyses to assess both transcriptional and translational levels. At the transcriptional level, the relative mRNA expression of IGF1, PI3K, AKT1, P27 and CCND2 was significantly lower in the MCT + RIV group than in the MCT group (*p* < 0.05) (Figure 9a). As shown in Figures 9b,c, the protein levels of p-PI3K/PI3K, p-AKT/AKT, CCND2, P27KIP1, and IGF1 were also significantly lower in the MCT + RIV group than in the MCT group (*p* < 0.05). These results indicate that rivaroxaban inhibits the PI3K/Akt signaling pathway in the livers of MCT-induced HSOS mice.

### 3.6 Spearman correlation coefficient analysis

We performed Spearman correlation coefficient analysis to investigate the correlation between the gut microbiota and serum biochemical analysis, as well as gene expression in small intestinal tissues and liver tissues. The analysis results are shown in Figure 9d. Among the gut microbiota mentioned above, *Firmicutes*, *Staphylococcaceae (including Staphylococcus),* and *Stenotrophomonas* have been positively correlated with PI3K pathway-related genes and serum biochemical indicators. *Streptococcaceae* and *Streptococcus* only showed a positive correlation with serum biochemical indicators. *Candidatus Arthromitus* only showed a positive correlation with PI3K pathway-related genes. However, *Actinobacteria*, *Adlercreutzia*, and *Oscillospira* showed a negative correlation with PI3K pathway-related genes and serum biochemical indicators. *Bacteroidetes* only have negative correlations with serum biochemical indicators. *Lactobacillus*, *Cyanobacteria*, and *Clostridium* only had negative correlations with PI3K-related genes. Interestingly, *TM7* had a positive correlation with PI3K pathway-related genes but a negative correlation with serum biochemical indicators. *Acinetobacter* showed a negative correlation with PI3K pathway-related genes but a positive correlation with AST (*p* < 0.05, |R| > 0.5). In addition, *Ochrobacterium* had a positively correlated with PI3K pathway-related genes and serum biochemical indicators. *Roseburia* had a positively correlated with PI3K pathway-related genes. *Bilophila*, *Streptomyces*, and *Blautia* showed a positive correlation with serum biochemical indicators. However, *Corynebacterium*, *Alistipes*, *Rhodococcus*, and *Ralstonia* are negatively related to PI3K pathway-related genes. Tenericutes and *Butyricicoccus* were negatively correlated with serum biochemical indicators. *cc_115*, *Brevundimonas*, *Silene* had positively correlated with PI3K pathway-related genes, but negatively correlated with serum biochemical indicators (*p* < 0.05, |R| > 0.5).

### 3.7 Gut microbiota drives the protective effect of rivaroxaban on MCT-induced HSOS

To determine whether the protective effect of rivaroxaban against MCT-induced HSOS is mediated via modulation of the gut microbiota, we performed both reverse and forward validation experiments. In the reverse validation, the gut microbiota was depleted via an antibiotic (ABX) cocktail, whereas in the forward validation, fecal microbiota transplantation from rivaroxaban-treated mice (FMT-RIV) was conducted. The results demonstrated that, in MCT-HSOS mice pretreated with ABX, rivaroxaban failed to improve the dark-red liver appearance (Figure 2b), the results of serum liver function biochemical analysis (Figure 2c), and hepatic histopathological features, including central venule endothelial cell injury, coagulative necrosis of hepatocytes, subendothelial hemorrhage of the central vein, hepatic sinusoidal hemorrhage, and hepatic fibrosis (Figure 2d). In contrast, these pathological phenotypes were markedly alleviated following FMT-RIV treatment (Figures 2f–h), suggesting that the protective effect of rivaroxaban against HSOS is at least partially dependent on its ability to modulate the gut microbiota.

## 4 Discussion

HSOS is associated with HSCT and pyrrolizidine alkaloids, and research strongly supports the effectiveness of anticoagulation for HSOS (Yang et al., 2019). Rivaroxaban, an oral anticoagulant, is prescribed for the clinical prevention and treatment of stroke in nonvalvular atrial fibrillation, deep vein thrombosis, and pulmonary embolism (Ajmal et al., 2021). However, to the best of our knowledge, there have been no reports worldwide on the effects of rivaroxaban on HSOS. Therefore, this study focused on exploring the therapeutic impact of rivaroxaban on HSOS and its underlying mechanisms. We investigated the effects of rivaroxaban on serum liver function, histopathology, the gut microbiota, and the molecular mechanism of MCT-HSOS in C57BL/6 mice. To determine the appropriate dose for mice, we referred to clinical dosing standards. The recommended initial treatment dose of rivaroxaban for acute deep vein thrombosis (DVT) during the first 3 weeks of therapy in clinical practice is 30 mg/day (Bauersachs et al., 2010). Based on body surface area conversion, this human dose corresponds to approximately 6.18 mg/kg/day in mice. Therefore, we selected 6.18 mg/kg/day as the intervention dose of rivaroxaban in our study. Our findings demonstrated that rivaroxaban effectively alleviated MCT-HSOS in C57BL/6 mice, possibly through two independent mechanisms: modulation of the gut microbiota and inhibition of the PI3K/Akt signaling pathway.

The present study revealed that rivaroxaban alleviated serum liver function in MCT-HSOS mice. This result coincides with previous research demonstrating that rivaroxaban alleviates CCl4-induced liver function injury (Mahmoud et al., 2019). We utilized H&E and Masson staining to evaluate liver histopathology injury, and the results revealed that rivaroxaban effectively reduced central venule endothelial cell injury, coagulative necrosis of liver cells, subendothelial hemorrhage of the central vein, hepatic sinusoidal hemorrhage and perihepatocellular fibrosis in the liver tissue of MCT-HSOS mice. This research represents the first report on the efficacy of rivaroxaban in alleviating MCT-HSOS. However, the exact mechanisms responsible for this effect remain to be fully understood.

A growing body of studies has demonstrated that the gut microbiota plays a significant role in liver disease. Thus, we investigated the impact of rivaroxaban on the gut microbiota of MCT-HSOS mice. Furthermore, ABX and FMT experiments demonstrated that the protective effect of rivaroxaban against HSOS is mediated through its regulation of the gut microbiota. The abundance of *Firmicutes* increased at the phylum level, but the abundances of *Bacteroidetes* and *Actinobacteria* decreased in the MCT group compared with those in the control group. A higher Firmicutes/Bacteroidetes (F/B) ratio has been reported in individuals with diseases such as obesity, non-alcoholic fatty liver disease (NAFLD), hyperglycemia, and alcoholic liver disease (ALD) (Riva et al., 2017; Porras et al., 2017; Lefever et al., 2016; Wang et al., 2019). Our results are in agreement with this observation. The four diseases mentioned above are associated with disruptions in lipid metabolism.

Moreover, the GO analysis revealed that the DEGs in the MCT group were significantly enriched in terms related to lipid metabolism compared with those in the control group. Therefore, the increased F/B ratio in our study may be related to lipid metabolism. Hepatic sinusoidal endothelial cell (SEC) injury is the primary pathogenic cause of HSOS (Yang et al., 2019). Research on atherosclerosis has shown that low-density lipoprotein tends to accumulate excessively in the endothelial cells of blood vessels (Baumer et al., 2017). This accumulation can cause endothelial dysfunction and injury by triggering inflammation (Liu et al., 2020). This finding suggests that the gut microbiota may contribute to the development of HSOS by damaging the SEC through disruptions in lipid metabolism. Additionally, *Actinobacteria* are associated with intestinal permeability, the immune system, metabolism, and the gut-brain axis (Binda et al., 2018). Research has demonstrated that *Actinobacteria* and *Bifidobacterium* suppress the inflammatory reaction in mice stimulated with alcohol or a high-fat diet (Yi et al., 2020). Our results revealed that the MCT group presented a reduced abundance of *Actinobacteria*, which may have contributed to the inflammatory response observed in HSOS. These results indicate that the gut microbiota of MCT-HSOS mice is dysbiotic at the phylum level.

At the genus level, LEfSe revealed greater abundances of *Streptococcus* and *Staphylococcus* in the MCT group than in the other three groups. There was a greater abundance of *Candidatus Arthromitus* in the MCT group than in the MCT + RIV group. Zhong *et al*. reported that *Streptococcus* could predict the extent of liver damage in patients with ALD (Zhong et al., 2021). Qin et al. reported that patients with liver fibrosis had a greater abundance of *Streptococcus* in their gut microbiota compared to healthy individuals (Qin et al., 2014). Both of these studies support our findings. *Streptococcus pyogenes* can activate the host inflammasome through its surface M protein. This activation can lead to pyroptotic host cell death and the maturation of proinflammatory cytokines, including IL-1β and IL-18 (Cunningham, 2017). *Staphylococcus aureus*, a member of the *Staphylococcaceae* family, is a prevalent pathogenic bacterium that can induce toxic and immunogenic responses (Sun et al., 2019). In addition, toxic shock syndrome toxin-1 (TSST-1), produced by *Staphylococcus aureus*, and pyrogenic exotoxins, produced by *Streptococcus pyogenes,* exhibit the properties of superantigens. These gram-positive exotoxins specifically attach to the major histocompatibility complex class II (MHC II) and restrict the T-lymphocyte receptor (TCR) VB domain. This interaction leads to the activation of a significant proportion of T cells, resulting in the subsequent release of proinflammatory lymphokines (Cohen, 2002). Moreover, harmful chemicals produced by these bacteria can infiltrate the liver via the portal vein, causing liver damage (Szabo, 2015). *Candidatus Arthromitus* has a significant effect on the immune system by activating MHC II, stimulating the T-cell response, and promoting the development of Th17 cells (Caselli et al., 2010; Ivanov et al., 2009). In summary, compared to the MCT + RIV group, the MCT group exhibited an increase in *Streptococcus*, *Staphylococcus*, and *Candidatus Arthromitus*, which may contribute to the development of HSOS by stimulating inflammation, releasing harmful compounds, and modulating the immune system.

On the other hand, rivaroxaban has the potential to inhibit these reactions by modulating the gut microbiota. However, our data did not clarify the precise mechanisms by which rivaroxaban modulates the gut microbiota. Excessive activation of the inflammasome may drive the gut microbiota toward a proinflammatory phenotype (Pellegrini et al., 2020). Ito and Li et al. reported that rivaroxaban effectively reduced inflammasome formation in studies related to atherosclerosis and cardiovascular complications of type 2 diabetes (Ito et al., 2021; Li et al., 2022). In HSOS, injury to sinusoidal endothelial cells results in loss of wall integrity and sinusoidal obstruction, ultimately leading to postsinusoidal portal hypertension (PH) (Bonifazi et al., 2020). Portal hypertension-induced structural changes in the intestinal epithelial layer play a key role in bacterial translocation (Trebicka et al., 2021). Vilaseca et al. demonstrated that rivaroxaban effectively lowers portal pressure in cirrhotic rats by decreasing O₂^−^ and improving NO bioavailability, deactivating hepatic stellate cells, and reducing fibrin deposition (Vilaseca et al., 2017). Therefore, by lowering portal hypertension in HSOS, rivaroxaban may indirectly mitigate bacterial translocation associated with structural changes in the intestinal epithelial layer. Further investigations are needed to explore the precise processes involved.

The molecular mechanism by which rivaroxaban affects HSOS remains unknown. The “gut-liver axis” is a two-way communication system between the gut microbiota and the liver. Our study demonstrated, through ABX and FMT experiments, that rivaroxaban exerts hepatoprotective effects against MCT-induced HSOS via the gut-liver axis. This connection is mostly established through the portal vascular system and is typically associated with the gut microbiota, mucus barrier, epithelial barrier, and antimicrobial peptides. This axis plays a crucial role in regulating the development of NAFLD and ALD (Albillos et al., 2020). To examine how rivaroxaban affects the “gut-liver axis,” we conducted a transcriptomic analysis of small intestinal tissue and validated the results via real-time qPCR and Western blot analyses of liver tissues. Our results indicated that rivaroxaban regulated the PI3K/Akt signaling pathway in small intestinal tissue and effectively suppressed it in the liver tissue of MCT-HSOS mice. Previous studies revealed that the PI3K/Akt signaling pathway is involved in the development of MCT-HSOS (Zheng et al., 2016; Zhang et al., 2017). PI3Ks are categorized into three classes, with class I PI3Ks being accountable for generating phosphatidylinositol (3,4,5)-trisphosphate (PIP3). PIP3 can bind to the pleckstrin homology (PH) domain of Akt and phosphoinositide-dependent protein kinase 1 (PDK1), leading to the phosphorylation and activation of Akt. Activated Akt translocates to the cytoplasm and nucleus, playing a role in various cellular processes (Zeng et al., 2012).

Additionally, the PI3K/Akt signaling pathway is involved in the progression of several liver disorders. For example, hyperglycemia exacerbates liver damage from acetaminophen by stimulating the PI3K/Akt signaling pathway, which promotes oxidative stress and triggers an inflammatory response in Kupffer cells (Wang et al., 2019). Moreover, the PI3K/Akt signaling pathway is strongly correlated with the activation of hepatic stellate cells, which in turn facilitates liver fibrosis (Ji et al., 2021). Conversely, maltol inhibits the PI3K/Akt signaling pathway, triggers apoptosis in activated hepatic stellate cells, and reduces the severity of liver fibrosis (Mi et al., 2019). Notably, the PI3K/Akt signaling pathway is significantly associated with lipid metabolism. The activation of the PI3K/Akt/mTOR pathway stimulates lipid synthesis, hence promoting the development of NAFLD-related HCC (Chen et al., 2019). However, Hu et al. suggested that D-mannose modulates the lipid metabolism of hepatocytes to relieve alcoholic liver disease (ALD) by suppressing the PI3K/Akt/mTOR pathway (Hu et al., 2022). Curiously, Ana Silva et al. and Chen et al. reported a decrease in the expression of P27 when the PI3K/Akt signaling pathway was activated, contrary to our results (Silva et al., 2021; Chen et al., 2021). This discrepancy may be attributed to additional variables that regulate P27 expression. Previous research has shown that MYC and PIM downregulate P27 expression, whereas FOXO and MENIN upregulate P27 expression (Hnit et al., 2015), suggesting that rivaroxaban effectively mitigates HSOS by inhibiting the PI3K/Akt signaling pathway. This inhibition likely involves the regulation of Kupffer cells and anti-inflammatory, antifibrotic, and lipid metabolism functions. However, further investigations are needed to determine the exact role of these functions.

Correlation analysis revealed that *Candidatus Arthromitus* and *Stenotrophomonas* were positively correlated with genes related to the PI3K pathway. In contrast, *Lactobacillus and Clostridium* were negatively correlated with genes related to the PI3K pathway. Limited studies have indicated that oral administration of *Lactobacillus* inhibits the PI3K/Akt signaling pathway by preventing Akt phosphorylation (Jin-Ju et al., 2015). Given that rivaroxaban regulates the PI3K/Akt signaling pathway in small intestinal tissue and effectively suppresses it in the liver tissue of MCT-HSOS mice, and on the basis of the results of correlation analysis, rivaroxaban is proposed as a potential treatment for HSOS by modulating the gut microbiota to inhibit the PI3K/Akt signaling pathway. However, our study does not provide sufficient evidence to establish a causal relationship between gut microbiota alterations and modulation of the PI3K/Akt signaling pathway under rivaroxaban treatment in MCT-HSOS mice. Further studies are warranted to elucidate the underlying mechanisms involved.

## 5 Conclusion

This study demonstrated that rivaroxaban significantly ameliorated hepatic injury in MCT-HSOS mice independently through modulation of the gut microbiota composition and inhibition of the PI3K/Akt signaling pathway. Specifically, rivaroxaban increased the relative abundance of *Allobaculum* and *Pediococcus*, but decreased the relative abundance of *Streptococcus*, *Staphylococcus*, and *Candidatus Arthromitus* in MCT-HSOS mice. Correlation analysis further revealed a potential link between the observed alterations in the gut microbiota and the inhibition of the PI3K/Akt pathway in this model. However, the current data are insufficient to elucidate the precise molecular mechanisms by which rivaroxaban influences the gut microbiota and the PI3K/Akt pathway or to determine whether these two effects are interdependent. This limitation highlights the need for further mechanistic investigations. Thus, this study offers a novel therapeutic approach for the clinical management of HSOS.

## Data Availability

The data presented in the study are deposited in the NCBI Sequence Read Archive (SRA) repository, accession numbers PRJNA1303002 and PRJNA1302683.

## Footnotes

^1^ http://kiwi.cs.dal.ca/Software/STAMP

^2^ http://www.personalbio.cn/

^3^ https://www.genescloud.cn
